# Anterior Chamber Angle in Adults Born Extremely, Very, and Moderately Preterm with and without Retinopathy of Prematurity—Results of the Gutenberg Prematurity Eye Study

**DOI:** 10.3390/children9020281

**Published:** 2022-02-18

**Authors:** Achim Fieß, Sandra Gißler, Eva Mildenberger, Michael S. Urschitz, Agnes Fauer, Heike M. Elflein, Fred Zepp, Bernhard Stoffelns, Norbert Pfeiffer, Alexander K. Schuster

**Affiliations:** 1 Department of Ophthalmology, University Medical Center of the Johannes Gutenberg University Mainz, 55131 Mainz, Germany; sandra.gissler@unimedizin-mainz.de (S.G.); agnes.fauer@unimedizin-mainz.de (A.F.); heike.elflein@unimedizin-mainz.de (H.M.E.); bernhard.stoffelns@unimedizin-mainz.de (B.S.); norbert.pfeiffer@unimedizin-mainz.de (N.P.); alexander.schuster@unimedizin-mainz.de (A.K.S.); 2 Division of Neonatology, Department of Pediatrics, University Medical Center of the Johannes Gutenberg University Mainz, 55131 Mainz, Germany; eva.mildenberger@unimedizin-mainz.de (E.M.); zepp@mail.uni-mainz.de (F.Z.); 3 Division of Pediatric Epidemiology, Institute for Medical Biostatistics, Epidemiology and Informatics, University Medical Center of the Johannes Gutenberg University Mainz, 55131 Mainz, Germany; urschitz@uni-mainz.de

**Keywords:** anterior chamber angle, anatomy, epidemiology, prematurity, retinopathy of prematurity

## Abstract

Purpose: To determine whether prematurity and associated factors affect the anterior chamber angle (ACA) width in adulthood. Methods: The Gutenberg Prematurity Eye Study (GPES) is a retrospective cohort study with a prospective ophthalmologic examination of adults (age 18–52 years) in Germany. All participants were examined with Scheimpflug imaging (Pentacam HR, Oculus Optikgeräte GmbH, Wetzlar, Germany) using linear regression analysis to assess the associations of ACA in the different sectors with gestational age (GA), birth weight, birth weight percentile, retinopathy of prematurity (ROP), ROP treatment, placental insufficiency, preeclampsia, and breastfeeding. Results: In total, 516 eyes of 319 preterm and full-term individuals (aged 28.9 ± 8.8 years, 188 females) were examined. ROP treatment was associated with smaller ACA width in the nasal (B = −9.6 [95%CI: −14.7; −4.5] degree; *p* < 0.001) and temporal positions (B = −11.5 [95%CI: −17.7; −5.3] degree; *p* = 0.001), whereas non-treated individuals with ROP had an unaltered ACA width, as did individuals with low gestational age without ROP. Conclusion: Advanced stages of ROP following treatment with laser- and cryocoagulation lead to a smaller ACA width until adulthood, and hence may increase the risk of angle closure in later life.

## 1. Introduction

Prematurity, low birth weight, and the postnatal occurrence of retinopathy of prematurity (ROP) are associated with altered ocular anatomic development in childhood. Children born preterm have altered corneal shape [1,2,3,4], shallower anterior chamber depth [1,3], increased lens thickness [1], and shorter axial length [2,3]. Furthermore, in a recent population-based report of adults aged 40 to 80 years, a steeper and thinner cornea, a smaller corneal diameter, and shorter axial length were reported in individuals born with a low birth weight (<2500 g) [5]. The combination of these various morphologic alterations may contribute to a narrowing anterior chamber angle, which may have a clinical impact on ocular morbidities, such as angle-close.

Several factors are associated with anterior chamber angle (ACA) width in adults, namely gender, age, corneal curvature, and refractive state [6]. However, there is a lack of information about the influence of prematurity on ACA width, with only a few reports analyzing ACA configuration in children born preterm with ROP [7,8,9,10]. Two studies assessed anterior chamber structures with gonioscopy, reporting a small ACA width in individuals born preterm with ROP [7,8]. In contrast, Cernichiaro-Espinosa et al. [9] used a spectral-domain optic coherence tomography (SD-OCT) device to measure the iridocorneal width of the ACA, observing a larger angle opening in children with ROP younger than one year compared with age-matched controls. In an SD-OCT study of Lenis et al. [11], the authors observed ACA narrowing particularly in seven ROP-treated children compared with five full-term children aged 9 years. In another study, ACA width, as measured with Scheimpflug imaging, was not different in children born preterm compared with individuals born at term aged between 7 and 14 years. However, this study included a heterogeneous group of individuals born preterm with and without ROP and did not stratify ROP [10]. To date, no data exist on the ACA width of adults born preterm, which may contribute to the increased ocular morbidities associated with smaller ACA width in adulthood. Hence, this investigation aimed to analyze the differences in ACA width in individuals born preterm with different degrees of prematurity with and without ROP. It was hypothesized that prematurity is related to a smaller ACA width in adulthood.

## 2. Methods

### 2.1. Study Population

The Gutenberg Prematurity Eye Study (GPES) is a single-center cohort study at the University Medical Center of the Johannes Gutenberg University Mainz in Germany (UMCM) that recruits individuals that (i) were born preterm or at term between 1969 and 2002 and (ii) were between 18 and 52 years of age at study enrolment. According to these design elements, the study is a retrospective cohort study with a prospective acquisition of follow-up data. Every preterm newborn with gestational age at birth (GA) ≤ 32 weeks and every second randomly chosen preterm newborn with GA 33–36 weeks was contacted and invited to participate in the study. Three male and three female randomly selected full-term subjects with a birth weight between the 10th and 90th percentile were also invited to serve as controls for each calendar month (from 1969 to 2002).

**Figure 1:**
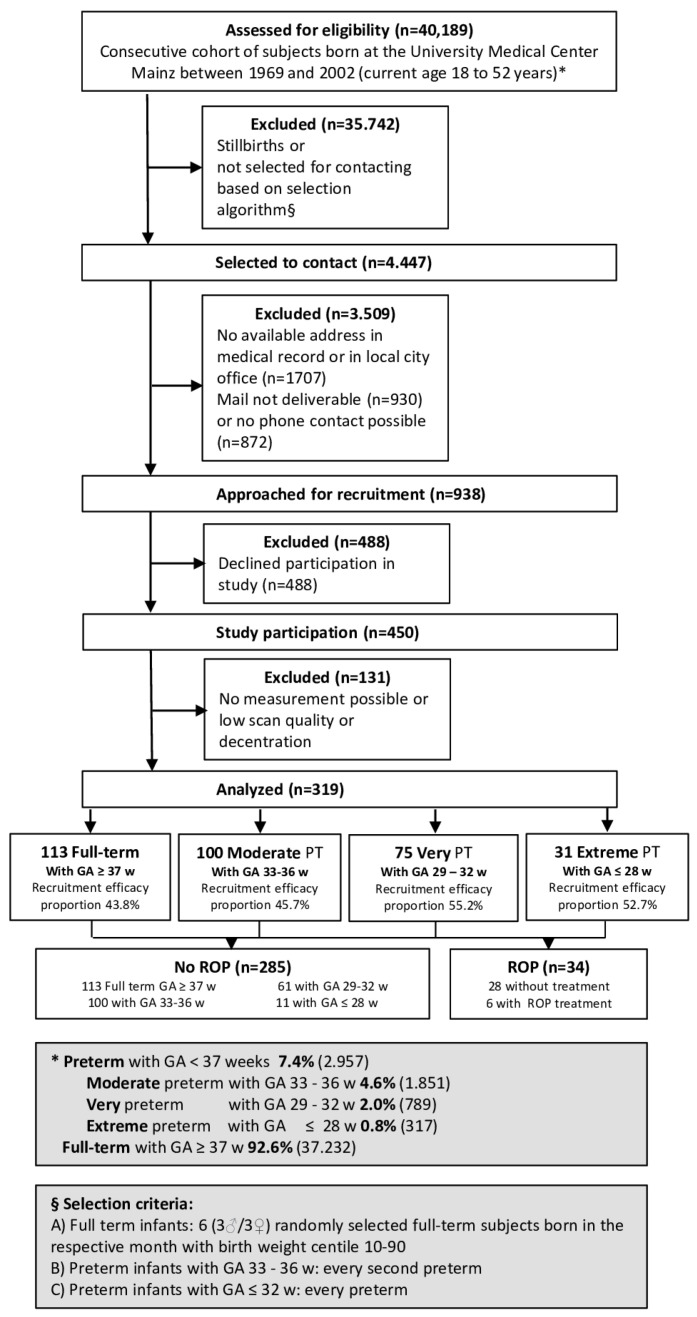
Study design of the Gutenberg Prematurity Eye Study. PT—preterm, GA—gestational age.

The study examinations were performed between 2019 and 2021 and the flow chart for eligibility and effective recruitment efficacy proportion is displayed in Figure 1. A detailed ophthalmological examination was conducted, including Scheimpflug imaging and a medical history interview. Furthermore, the medical records of the study participants documenting the perinatal and postnatal history were reviewed.

Written informed consent was obtained from all participants before enrolment. The GPES was carried out in conformity with Good Clinical Practice, Good Epidemiological Practice, and the ethical principles of the Declaration of Helsinki. The research project was supported by the local ethics commission of the Medical Chamber of Rhineland-Palatinate, Germany (reference no. 2019-14161; original vote: 29 May 2019, latest update: 02 April 2020).

### 2.2. Assessment of Pre-, Peri- and Postnatal Medical History

The participants’ medical history was assessed from their medical files stored at the UMCM to collect data regarding GA (weeks), birth weight (kg), diagnosis of ROP, stage of ROP and ROP treatment, placental insufficiency, preeclampsia, and breastfeeding. Birth weight percentiles were calculated according to Voigt et al. [12].

### 2.3. Categorization

For descriptive analysis, participants were grouped into full-term participants (GA ≥ 37 completed weeks) (group 1), preterm participants with GA between 33 and 36 weeks without ROP (group 2), preterm participants with GA between 29 and 32 weeks without ROP (group 3), preterm participants with GA ≤ 28 weeks without ROP (group 4); and preterm participants with GA ≤ 32 with the postnatal presence of ROP, without ROP treatment (group 5) and with ROP treatment (group 6). In the case that only one eye of a participant had ROP, the other non-ROP eye was excluded from the analysis.

### 2.4. Ophthalmologic Examination

A comprehensive ophthalmologic examination was conducted consisting of testing visual acuity and refraction (ARK-1s, NIDEK, Oculus, Wetzlar, Germany) and intraocular pressure measurement with a non-contact tonometer (NT 2000™, Nidek Co., Tokyo, Japan). Scheimpflug imaging was performed using a Pentacam™ instrument (Oculus, Wetzlar, Germany) and biometry measurement with a Lenstar LS900 (Haag-Streit, Bern, Switzerland).

### 2.5. Scheimpflug Imaging

A Pentacam HR rotating Scheimpflug camera was used for detailed tomography of the anterior eye enabling three-dimensional tomography. Each examination was conducted in congruence with strict standardized SOPs to reduce examiner-dependent variance. When optimal alignment was provided, Scheimpflug imaging was performed while participants had to fixate on a fixation light. Within every Scheimpflug measurement, 25 Scheimpflug images were captured for the anterior segment and all scans and quality descriptions of the device were checked. The nasal and temporal ACA were calculated by the device and participants were only included in the present analysis when nasal or temporal ACA width measurements were available. Each of the 25 scans was reviewed and checked. If ACA angle measurements were invalid, these scans were excluded. ACA width measurements were also controlled for outliers, and in the case of a suspected artefact or invalid measurement, removed. Superior and inferior ACA width were not included because these measurements are frequently invalid due to an overlapping upper or lower lid.

### 2.6. Covariates

The factors considered to affect the main outcome measures include GA (weeks), birth weight (kg), birth weight percentile, ROP (yes), ROP treatment (yes), placental insufficiency (yes), preeclampsia (yes), and breastfeeding (yes). Participants with a history of corneal or cataract surgery were excluded as this may have contributed to altered ocular anatomy.

### 2.7. Statistical Analysis

The main outcome measures were the ACA width of the nasal and temporal sectors. Descriptive statistics were computed for ACA width stratified by clinical group. For dichotomous parameters, absolute and relative frequencies were calculated. The mean and standard deviation were computed for approximately normally distributed variables, otherwise median and interquartile range. Linear regression models with general estimating equations (GEE) were used to calculate associations and to account for correlations between two eyes of one study participant. Because of the low number of participants in group #6, a Mann–Whitney U test was conducted to compare ACA width with the control group. Univariate analyses between the main outcome measures and GA (weeks), birth weight (kg), birth weight percentile, ROP (yes), ROP treatment (yes), placental insufficiency (yes), preeclampsia (yes), and breastfeeding (yes) were computed. The present investigation was an explorative study and no adjustment for multiple testing was performed. Analyses were conducted with commercial software (IBM SPSS 20.0; SPSS, Inc., Chicago, IL, USA).

## 3. Results

### 3.1. Participant Characteristics

In the present analysis, 516 eyes of 319 preterm and full-term individuals (aged 28.9 ± 8.8 years, 188 females) were included. Overall, 182 eyes of 113 participants with GA ≥ 37 weeks (group 1), 160 eyes of 100 participants with a GA between 33 and 36 weeks without ROP (group 2), 101 eyes of 61 participants with a GA between 29 and 32 weeks without ROP (group 3), 18 eyes of 11 participants with a GA ≤ 28 weeks without ROP (group 4), 45 eyes of 28 participants with a GA between 24 and 32 weeks with ROP without treatment (group 5), and 10 eyes of 6 participants with a GA between 24 and 32 and with postnatal treatment for ROP were assessed (group 6). In the group with ROP treatment, three participants (five eyes) underwent laser coagulation and three participants (five eyes) underwent cryocoagulation (Table 1). The recruitment efficacy proportion for each group is presented in Figure 1. Overall, 131 participants were excluded because Scheimpflug imaging was not possible, ACA width segmentation errors occurred, or cataract surgery had been conducted. Furthermore, eight eyes without ROP were excluded in which the fellow eye had postnatal ROP.

### 3.2. Descriptive Ocular Geometric Parameters

**Figure 2:**
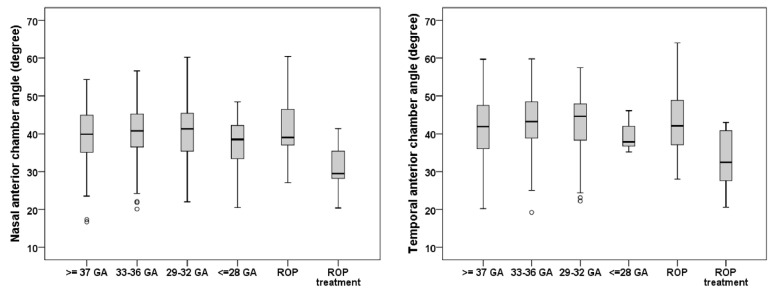
Boxplots of the nasal and temporal anterior chamber angle widths of the different study groups. The ROP-treated group showed the smallest nasal and temporal anterior chamber angles (n = 319). ROP—retinopathy of prematurity, GA—gestational age.

Smaller nasal (n = 10 eyes) and temporal (n = 10 eyes) ACA widths were descriptively observed in individuals born preterm with postnatal treatment for ROP compared with the control group assessed with Mann–Whitney U test. The nasal and temporal ACA width was comparable between the full-term control group and the preterm groups with and without ROP (Table 2). The boxplots in Figure 2 present the nasal and temporal ACA widths for the different groups, with the ROP-treated group demonstrating the smallest nasal and temporal ACA widths in this study.

### 3.3. Association Analysis

In univariate analysis, a narrower ACA in the nasal (B = −9.6 [95%CI: −14.7; −4.5]; degree *p* < 0.001) and temporal position (B = −11.5 [95%CI: −17.7; −5.3] degree; *p* = 0.001) was associated with ROP treatment, but neither with postnatal ROP occurrence nor gestational age, birth weight, birth weight percentile, preeclampsia, breastfeeding and placental insufficiency (Table 3).

## 4. Discussion

This study provides the first data about the association of prematurity and associated factors with ACA width in adulthood. Individuals born preterm with postnatal advanced ROP stages and following treatment revealed a smaller ACA width in the nasal and temporal sector compared with individuals born full-term. Furthermore, this study shows that low birth weight, preterm delivery, and lower ROP stages without need for ROP treatment do not alter the configuration of the ACA width, while advanced ROP stages following treatment with cryo- or laser-therapy are associated with a shallower ACA. This may be of clinical importance because a narrow ACA is a known risk factor for angle closure.

Until recently, gonioscopy was used as the standard for measuring ACA width [13,14], but new quantitative imaging modalities have been developed, allowing detailed anterior segment measurement using ultrasound biomicroscopy, optical coherence tomography, and Scheimpflug imaging. However, only optical coherence tomography and Scheimpflug imaging allow clinicians to obtain a quantitative and rapid non-contact measurement of the anterior segment and ACA configuration, making this method attractive for the examination of adults [15] and children [10].

There are only a few reports examining the ACA structures of individuals born preterm in the literature. Chang et al. [8] used gonioscopy to examine 54 eyes of 29 children (mean age of 9.3 ± 2.4 years) born preterm with ROP stage I, II, III, and postnatal laser treatment, reporting a narrowing ACA in the superior, nasal, and temporal quadrants in children born preterm with ROP compared with 134 eyes of 67 children born at term. In addition, they observed a steeper iris curvature and a more anteriorly curved and inserted iris compared with children born at term. Hartnett et al. [7] investigated 26 eyes with gonioscopy of infants born preterm (age 0.9 years) with ROP stage IV or V having received no ROP treatment, observing an angle closure greater than 180 degrees in 12% of participants, and 58% had a high iris convexity. In contrast, Cernichiaro-Espinosa et al. [9] examined ACA structures with SD-OCT in 27 eyes of 14 children with ROP (mean age at examination of 0.3 years) and compared their findings with 21 eyes of 13 children without ROP. The authors reported a larger angle opening of 37.3° in the ROP group and of 34.7° in the no ROP group. Marginally lower ACA width measurements than the present study were reported by Ecsedy et al. [10], also using Scheimpflug imaging. Ecsedy et al. [10] investigated 50 eyes of 27 individuals born preterm with and without ROP and compared them with an age-matched control group, showing that the mean ACA width for the preterm group (34.6 ± 19°) was comparable to the full-term group (35.6 ± 8°). Senthil et al. [11] observed, in a very small study group containing seven ROP laser-treated and five control children born at term, smaller ACA width in the ROP-treated group measured by OCT. Quinn et al. [16] randomized in the CRYO-ROP study the two eyes of each study patient to either cryo-coagulation or no treatment and found no difference in subsequent myopia development, which in preterms is mostly due to anterior segment dysgenesis. The present study provides new data that advanced ROP stage with following treatment results in a narrower ACA configuration, while other perinatal parameters show no effect.

Previous authors reported that prenatal ACA formation takes until the eighth month of gestation. At the time of birth, there is a translucent membrane that then widens during the first years of life. Accordingly, it is of interest whether ACA developmental alterations are caused by low birth weight, prematurity, and ROP, and whether alterations persist until adulthood, contributing to an increased risk for ocular disease. Fiedler et al. hypothesized that a decisive factor influencing anterior segment development in preterm children may be the lower extrauterine temperature, probably leading to a developmental delay of the anterior segment [17]. In contrast to this hypothesis, we showed in our large cohort that prematurity and low birth weight do not contribute to ACA narrowing.

Chang et al. [8] suggested that a narrowing ACA and anteriorly curved iris may affect aqueous drainage, potentially leading to an increased risk of glaucoma development. This might be one reason, among others, contributing to the previously reported increased risk for secondary childhood glaucoma in advanced ROP stages with the need for ROP treatment [18]. The mean anterior chamber angle in the nasal and temporal position was about 10 degrees smaller in the group with ROP treatment than in the other study groups, indicating that anterior chamber angle narrowing in advanced ROP stages with need for treatment lasts until adulthood.

## 5. Strengths and Limitations

The study was limited due to the single-center and hospital-based study design. Furthermore, as individuals born extremely preterm are at an increased risk for poorer fixation, this could have affected the ACA width measurements, even though these measurements were quality controlled. Missing contact details and declination to take part in this study are other restrictions, although the recruitment efficacy proportion was acceptable (see Figure 1). It is noteworthy that the number of participants with ROP treatment was rather small, and this must be considered when interpreting our results. This is especially true for study participants with advanced ROP and treatment that suffer from nystagmus, loss of vision, or other non-ocular disabilities that did not allow a valid examination to be performed, and thus may lead to selection bias and a small number of included subjects in this group. Therefore, a future multicenter study should be conducted to replicate our findings. Furthermore, as advanced stages of ROP needed treatment, we cannot differentiate whether the advanced stage of ROP or the treatment (laser or cryotherapy) leads to the narrowing of ACA. With the recent approval of anti-VEGF therapy as ROP treatment, this might be possible to investigate in the future. Other quantitative parameters of the ACA, such as angle opening distance and trabecular–iris space area, were not available in the Scheimpflug system used; therefore, they could not be integrated into the analysis. As previously reported, full visualization of the entire angle cannot be obtained with Scheimpflug imaging [19]. As gonioscopy was not performed within the GPES, it was not possible to compare the present results with previous gonioscopic findings in the literature. As most of the individuals taking part in the GPES were Caucasian, conclusions should only be drawn for this ethnicity.

This study has numerous strengths. First, the sample size was large and the high proportion of subjects with ROP strengthens the analysis. Second, the large sample size of preterm individuals enables the scarce comparison between adults born at term with a high number of adults born preterm without ROP. Third, reviewing perinatal medical charts enabled a comprehensive analysis with the inclusion of several perinatal parameters potentially affecting ACA configuration. All measurements were performed according to strict standardized operating procedures to reduce examiner-dependent variation and every investigator was blinded to the participants’ birth history.

## 6. Conclusions

In conclusion, the present data analyses revealed a smaller ACA width in adults with previous ROP treatment, while those with ROP without treatment or those with low gestational age without ROP had similar ACA widths to the control group of individuals born at term. This finding indicates that advanced stages of ROP with ROP treatment may lead to a smaller ACA width in adults aged 18 to 52 years, which may impact the development of ocular diseases, such as angle closure.

## Figures and Tables

**Table 1:** Characteristics of the study sample (n = 319, eyes = 516) of the Gutenberg Prematurity Eye Study (GPES) stratified by study group.

	Group 1	Group 2	Group 3	Group 4	Group 5	Group 6
Gestational Age	GA ≥ 37	GA 33–36	GA 29–32	GA ≤ 28	GA ≤ 32	GA ≤ 32
		no ROP	no ROP	no ROP	ROP without Treatment	ROP with Treatment
Participants/eyes (n)	113/182	100/160	61/101	11/18	28/45	6/10
Sex (women) (%)	68 (60.2)	61 (61.0)	34 (55.7)	7 (63.6)	15 (53.6)	3 (50.0)
Age (y)	30.0 ± 9.2	30.2 ± 9.3	27.5 ± 7.7	23.5 ± 7.5	25.1 ± 6.1	25.2 ± 4.6
Birth weight (g)	3421 ± 389	2112 ± 479	1563 ± 337	931 ± 215	1068 ± 380	708 ± 143
Birth weight < 1500 g (yes)	0 (0%)	9 (9.0%)	25 (41.0%)	11 (100%)	24 (85.7%)	6 (100%)
Birth weight < 1000 g (yes)	0 (0%)	0 (0%)	4 (6.6%)	7 (63.6%)	14 (50.0%)	6 (100%)
Birth weight percentile	49.2 ± 20.8	27.1 ± 24.4	42.9 ± 25.3	51.1 ± 25.8	35.0 ± 25.1	26.2 ± 30.5
Gestational age (wks)	39.3 ± 1.3	34.3 ± 0.9	30.8 ± 1.1	26.3 ± 1.7	28.1 ± 2.0	26.0 ± 1.9
(min–max)	(37–43)	(33–36)	(29–32)	(23–28)	(24–32)	(24–32)
ROP stage (1/2/3)	0/0/0	0/0/0	0/0/0	0/0/0	16/25/4	0/3/7
Preeclampsia (yes)	8 (7.1%)	18 (18.0%)	8 (13.1%)	1 (9.1%)	6 (21.4%)	1 (16.7%)
Placental insufficiency (yes)	2 (1.8%)	10 (10.0%)	2 (3.3%)	0 (0%)	0 (0%)	0 (0%)
HELLP syndrome (yes)	0 (0%)	5 (5.0%)	0 (0%)	0 (0%)	3 (10.7%)	0 (0%)
Maternal smoking (eys)	5 (4.4%)	6 (6.0%)	6 (9.8%)	1 (9.1%)	2 (7.1%)	1 (16.7%)
Gestational diabetes (yes)	1 (0.9%)	5 (5.0%)	1 (1.6%)	0 (0.0%)	1 (3.6%)	0 (0%)
Breastfeeding (yes)	59 (52.2%)	55 (55.0%)	33 (54.1%)	4 (36.4%)	13 (46.4%)	2 (33.3%)
** Ocular parameters **						
Spherical equivalent (diopter) OD	−0.24 ± 2.58	−0.02 ± 3.15	0.16 ± 1.80	0.40 ± 1.26	0.50 ± 4.30	0.90 ± 2.40
Spherical equivalent (diopter) OS	−0.23 ± 2.60	−0.01 ± 3.10	0.17 ± 1.79	0.39 ± 1.24	0.49 ± 4.28	0.89 ± 4.38
Intraocular pressure (mmHg) OD	15.2 ± 2.8	14.7 ± 2.9	15.3 ± 3.3	16.6 ± 3.4	15.3 ± 4.3	16.8 ± 4.5
Intraocular pressure (mmHg) OS	15.1 ± 2.8	14.6 ± 2.8	15.2 ± 3.2	15.1 ±3.0	15.2 ± 4.2	16.7 ± 4.3

g—gram; mm—millimeter; GA—gestational age; ROP—retinopathy of prematurity; y—years; n—number; OD—right eye; OS—left eye.

**Table 2:** Anterior chamber angle of the different sectors of the GPES sample (n = 319) for each study group.

	Group 1	Group 2	Group 3	Group 4	Group 5	Group 6
Gestational Age	GA ≥ 37	GA 33–36	GA 29–32	GA ≤ 28	GA ≤ 32	GA ≤ 32
		no ROP	no ROP	no ROP	ROP without Treatment	ROP with Treatment
§ Participants/§eyes (n) OD + OS	** 113/182 **	** 100/160 **	** 61/101 **	** 11/18 **	** 28/45 **	** 6/10 **
** Anterior chamber angle **						
***Right eye***						
** Nasal (µm) OD **	40.1 ± 7.0	40.9 ± 7.0	40.7 ± 7.6	36.9 ± 4.7	42.5 ± 6.5	32.1 ± 6.3 **
Valid measurements OD (participants/eyes)	79/81	76/77	47/48	7/8	22/23	4/4
** Temporal (µm) OD **	41.8 ± 6.8	43.3 ± 6.8	43.0 ± 7.7	39.7 ± 3.0	42.5 ± 7.3	27.9 ± 0.4 **
Valid measurements OD (participants/eyes)	50/81	48/77	32/48	4/8	14/23	2/4
***Left eye***						
** Nasal (µm) OS **	39.0 ± 6.7	40.7 ± 6.5	40.1 ± 7.2	37.0 ± 8.6	40.0 ± 6.9	30.2 ± 6.9 **
Valid measurements OD (participants/eyes)	99/101	82/83	53/53	10/10	22/22	6/6
** Temporal (µm) OS **	40.4 ± 7.7	44.8 ± 7.3 #	42.1 ± 6.7	40.0 ± 3.7	44.4 ± 9.0	31.9 ± 9.0 #
Valid measurements OD (participants/eyes)	58/101	46/83	34/53	7/10	15/22	4/6

GA—gestational age; ROP—retinopathy of prematurity; µm—micrometer; OD—right eye; OS—left eye. § Participants/eyes were included if at least one measurement of the temporal OR nasal anterior chamber angle was possible. Linear regression analysis was applied to compare the different groups with the full-term control group (reference). ROP-treated eyes were compared with the control group with Mann–Whitney U test due to the low number of available measurements in group 6. # statistical difference (*p* < 0.05) compared with the control group. ** statistical difference (*p* < 0.001) compared with the control group.

**Table 3:** Linear associations of the temporal and nasal anterior chamber angles with perinatal parameters (n = 319) for GPES sample.

	Univariate Analysis	
	B (95% CI)	*p*
**Nasal anterior chamber angle** **(in degrees)**		
Breastfeeding	1.269 (−0.809; 3.348)	0.23
Placental insufficiency	−0.735 (−6.098; 4.629)	0.79
Preeclampsia	−1.098 (−3.175; 0.978)	0.30
Gestational age (weeks)	0.038 (−0.139; 0.214)	0.68
Birth weight (gram)	0.047 (−0.691; 0.784)	0.90
Birth weight percentile	0.000 (−0.030; 0.029)	0.97
ROP (yes)	−0.351 (−3.059; 2.357)	0.80
ROP treatment (yes)	−9.6 (−14.7; −4.5)	<0.001
**Temporal anterior chamber angle** **(in degrees)**		
Placental insufficiency (yes)	2.720 (−0.718; 6.158)	0.021
Preeclampsia (yes)	−1.211 (−4.320; 1.808)	0.43
Breastfeeding (yes)	1.039 (−0.941; 3.018)	0.30
Gestational age (weeks)	0.018 (−0.210; 0.247)	0.87
Birth weight (gram)	−0.249 (−1.242; 0.743)	0.62
Birth weight percentile	−0.015 (−0.056; 0.026)	0.47
ROP (yes)	−0.879 (−4.623; 2.864)	0.65
ROP treatment (yes)	−11.5 (−17.7; −5.3)	0.001

B—Beta; CI—confidence interval; mm—millimeter. Linear regression analysis using generalized estimating equations to control for correlations between right and left eyes.

## Data Availability

Achim Fieß had full access to all of the data in the study and takes responsibility for the integrity of the data and the accuracy of the data analysis. Statistical analyses were performed by A.F. The analysis presents the clinical data of a cohort. To meet the general idea of the verification and reproducibility of scientific findings, we offer access to data at the local database upon request at any time. Interested researchers should make their requests to the coordinating PI of the GPES (Achim Fieß; achim.fiess@unimedizin-mainz.de).
